# The Retina as a Model for Investigating Neurochemistry and Development in the Central Nervous System †

**DOI:** 10.3390/brainsci16070707

**Published:** 2026-06-30

**Authors:** Roberto Paes-de-Carvalho, Sriparna Majumdar

**Affiliations:** 1 Department of Neurobiology and Program of Neurosciences, Fluminense Federal University, Niterói 24210-201, RJ, Brazil; 2 Computer Science Department, City College of San Francisco, San Francisco, CA 94112, USA; smajumda@mail.ccsf.edu

## 1. Overview and Introduction

The retina is a thin layer of neural tissue at the back of the eye that detects visual stimuli and performs the first stage of processing information transmitted from the external environment. As part of the central nervous system, it has a highly organized layered structure in which cell bodies are separated from the neurite layers where synaptic connections form. Most, and perhaps all, neurotransmitters and neuromodulators identified in the brain are also found in the vertebrate retina. Among the neuroactive substances currently considered especially important are cannabinoids, interleukins, nitric oxide, and vitamin C [1,2,3,4]. Neurotrophins, such as NGF and BDNF, as well as peptides like PACAP, also have important functions in the central nervous system, including the retina, particularly during development. Vitamin C is highly concentrated in the brain and may also act as a neuromodulator in the retina [5]. Glutamate, the main excitatory neurotransmitter, is present at all synapses in the vertical pathway connecting photoreceptors, bipolar cells, and ganglion cells [6], whereas the inhibitory neurotransmitter GABA and dopamine are present in the lateral modulatory pathway formed by horizontal and amacrine cells [7].

Because of all these characteristics, the retina can be considered an excellent model for CNS neurochemical studies. The rat and chicken retinas are particularly useful models for studying CNS development because they can easily be dissected without contamination from other tissues over a rather long developmental period, allowing the collection of a large amount of tissue and the establishment of cultures in which many properties of the intact tissue are preserved [8]. During chicken retina development, metabotropic receptors for dopamine and the neuromodulator adenosine are expressed and coupled to adenylyl cyclase, generating either positive cyclic AMP signaling through dopamine D1-like and adenosine A2A receptors [9,10] or negative signaling through D2-like and adenosine A1 or A3 receptors [11,12,13].

Embryonic retinas can also be used to generate different culture types, including purified neuronal cultures, purified glial cultures, and mixed cultures containing both cell types [14,15,16]. This makes it possible to study neurons and glial cells separately, as well as to examine how they interact in mixed cultures.

This Special Issue therefore brings together important articles that highlight the value of the retinal model for studying neurotransmitters and other neuroactive substances in CNS neurochemistry and development.

## 2. Overview of Contributions

This Special Issue includes two reviews and three research articles.

(1) **Retinal Neurochemistry by Dominic Man-Kit Lam and George Ayoub**

This review provides an overview of the retinal cell types and neurotransmitters and explains how the complex CNS-like organization supports the transmission of visual information to other brain regions.

(2) **Ouabain Counteracts Retinal Ganglion Cell Death Through Modulation of BDNF and IL-1 Signaling Pathways by Amanda Candida da Rocha Oliveira et al.**

This article examines the effects of the steroid hormone ouabain, which binds to and inhibits Na^+^, K^+^-ATPase and is known for enhancing cardiac contractility. At low concentrations, ouabain can also activate signaling pathways in the CNS and protect retinal ganglion cells from axotomy-induced death in cultures of rat retinal cells. The findings point to the involvement of BDNF and TRKB receptor activation, as well as IL-1beta and the Src and PKC pathways.

(3) **Cannabinoids Activate Endoplasmic Reticulum Stress Response and Promote the Death of Avian Retinal Müller Cells in Culture by Ana Lúcia Marques Ventura et al.**

This article examines how cannabinoids induce glial cell death in retinal cultures. In cultures from chick embryonic retina, the CB1/CB2 agonist WIN 55,212-2 (WIN) produced substantial glial cell death. Similar effects were observed with cannabidiol (CBD), Δ^9^-tetrahydrocannabinol (THC), and CP55940, another CB1/CB2 agonist. CBD appears to act through CB2 receptors, because its effect was blocked by a CB2 receptor antagonist but not by a CB1 receptor antagonist. WIN also increased ROS production, JNK activation, and caspase-3 activity, while leaving p38 kinases unchanged. These effects were prevented by salubrinal, an inhibitor of elongation factor 2α dephosphorylation, indicating that cannabinoids promote retinal glial cell apoptosis through ROS generation, endoplasmic reticulum stress, JNK phosphorylation, and caspase-3 processing.

(4) **Vitamin C Modulates the PI3K/AKT Pathway via Glutamate and Nitric Oxide in Developing Avian Retina Cells in Culture by Aline Teixeira Duarte-Silva et al.**

This study of chicken retina cultures demonstrates that vitamin C increases extracellular glutamate levels, activates ionotropic glutamate receptors, and stimulates the PI3K/AKT pathway during retinal development. This effect depends on nitric oxide production and a calcium-dependent mechanism. The findings support an interaction between ascorbate and glutamate in the retina and further highlight the role of vitamin C as a neuromodulator in the central nervous system.

(5) **Disease Diagnosis Using Retinal Vasculature: Insights from Flammer Syndrome and AI by George Ayoub**

This review presents evidence of the interaction between the retina and the rest of the body, highlighting the value of retinal vascular analysis for several diseases. It also discusses the use of machine learning tools to interpret retinal images for monitoring treatment and disease progression.

## 3. Conclusions

This Special Issue presents different approaches that use the retina as a model for studying neurochemistry in the nervous system, with particular emphasis on neuromodulators and neuroactive substances, such as ouabain, neurotrophins, cannabinoids, and vitamin C. It also highlights the value of retinal cultures for investigating the actions of neurotransmitters and neuromodulators, as well as the retina’s potential as a window for analyzing different diseases. Because many studies in the literature follow this approach, further special issues on this topic could help integrate knowledge in this rapidly advancing field.
